# Recent Advances in Molecular Mechanisms of Nucleoside Antivirals

**DOI:** 10.3390/cimb45080433

**Published:** 2023-08-17

**Authors:** Polina N. Kamzeeva, Andrey V. Aralov, Vera A. Alferova, Vladimir A. Korshun

**Affiliations:** Shemyakin-Ovchinnikov Institute of Bioorganic Chemistry, Miklukho-Maklaya 16/10, 117997 Moscow, Russia; polinabast@yandex.ru (P.N.K.); baruh238@mail.ru (A.V.A.); alferovava@gmail.com (V.A.A.)

**Keywords:** nucleoside, antiviral, mechanism of action, activity, prodrug, viral polymerase, nucleotide, modification, nucleobase

## Abstract

The search for new drugs has been greatly accelerated by the emergence of new viruses and drug-resistant strains of known pathogens. Nucleoside analogues (NAs) are a prospective class of antivirals due to known safety profiles, which are important for rapid repurposing in the fight against emerging pathogens. Recent improvements in research methods have revealed new unexpected details in the mechanisms of action of NAs that can pave the way for new approaches for the further development of effective drugs. This review accounts advanced techniques in viral polymerase targeting, new viral and host enzyme targeting approaches, and prodrug-based strategies for the development of antiviral NAs.

## 1. Introduction

Over the past few years, humanity has become more aware of its vulnerability to the spread of new viruses and drug-resistant strains of known pathogens, as evidenced by the H1N1 influenza, Ebola, and the COVID-19 outbreaks/pandemic [1,2,3,4]. Viruses are obligate parasites that utilize the building blocks and translational machinery of host cells. Consequently, the viral genome encodes a minimal set of enzymes necessary for replication inside the cell [5]. This limits the available targets for the development of safe and effective therapeutic agents. Additionally, viruses exhibit a high mutation rate, leading to the development of drug resistance [6]. Therefore, the development of effective antiviral agents is a challenging and laborious task. However, amid the pandemic, when urgent actions are needed, repurposing existing drugs becomes an optimal approach [7]. The reduction in safety testing costs is among the advantages of this approach. The pursuit of repurposing drives the development of broad-spectrum antiviral agents. Therefore, the priority objectives in the field are reducing toxicity, expanding the spectrum of action, and overcoming resistance.

Nucleoside analogs (NAs) are of interest for the rapid and efficient development of antiviral agents. On the one hand, many factors influencing efficacy and safety profiles have already been studied, and more than 30 NAs have already been approved by the FDA [6,8,9]. On the other hand, an in-depth examination of NAs’ mechanisms of action (MoA) has revealed previously unknown details, which is attracting interest in the field and can be useful in the development of new, more efficient, and broadly acting agents. Many reviews dealing with nucleoside antivirals were published. Recent examples are accounts on fluorinated nucleosides [10,11], 4′-modified nucleosides [12], oxetanocin [13,14], marketed nucleosides [15], antiviral nucleosides and analogues [16,17,18,19,20,21,22,23,24,25,26,27,28,29,30,31,32,33,34,35], and the chemistry of nucleoside drugs [36,37,38]. However, these reviews predominantly consider chemical and structural aspects, with some discussion on the main nucleoside mechanisms of antiviral action [21,22,24,29,30,34,39]. This review will discuss approaches to the development of antiviral NAs, mentioning well-proven approaches and focusing on the latest findings on new antiviral mechanisms and targets for nucleoside-based agents.

## 2. Polymerase Targeting

**Figure 1:**
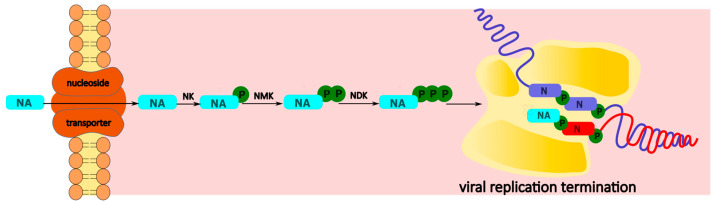
Classical mechanism of action of nucleoside analogues. NA—nucleoside analogue; NK—nucleoside kinase; NMK—nucleoside monophosphate kinase; NDK—nucleoside diphosphate kinase.

Traditionally, viral nucleic acid synthesizing enzymes (DNA polymerases, RNA dependent RNA polymerases, and reverse transcriptases) are the primary targets of inhibition of nucleoside-based drugs. To exert antiviral action, the NA enters the infected cell through a nucleoside transporter [40]. Subsequently, it is phosphorylated by viral and host cell kinases to the corresponding triphosphate (NA-TP). The latter is recognized as a substrate by viral polymerases and is incorporated into the viral nucleic acid. This incorporation ultimately leads to the inhibition of viral genome replication (Figure 1) [29]. However, the mechanisms by which the incorporated nucleotide derivative can inhibit viral genome synthesis are rather diversified.

### 2.1. Obligate Termination

**Figure 2:**
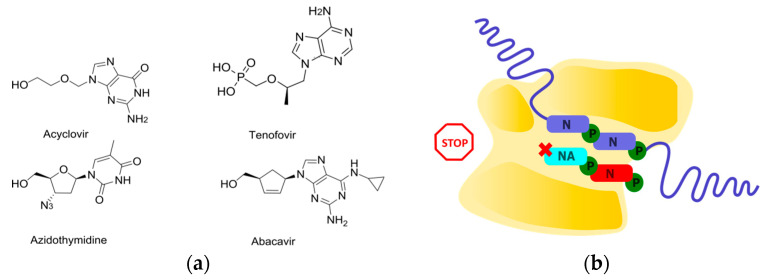
Obligate chain terminators. (**a**) Structures of FDA-approved obligate chain terminators. (**b**) Schematic representation of obligate chain termination.

The process of chain elongation involves the attachment of an incoming nucleoside triphosphate to the 3′-hydroxyl of the terminal nucleotide in the growing chain. Thus, the absence of this group makes it chemically impossible to attach the next nucleotide and continue the chain elongation. A class of NAs that lacks 3′-hydroxyl but is able to be incorporated into the chain can provide chain termination [21] and is referred to as obligate chain terminators. Within this concept, drugs have been developed to treat HSV, HCV, and HIV, including successful developments such as acyclovir, tenofovir, and abacavir (Figure 2) [21]. However, obligate chain terminators have several drawbacks. They are primarily used against DNA viruses and retroviruses. The effectiveness of obligate terminators against RNA viruses is generally low. Firstly, RNA-dependent RNA polymerases have evolved the ability to discriminate substrates based on the presence of a 2′-hydroxyl and have acquired some selectivity regarding the presence of a 3′-hydroxyl [41,42]. Secondly, incorporated NAs can be efficiently removed through pyrophosphorolysis [43].

**Figure 3:**
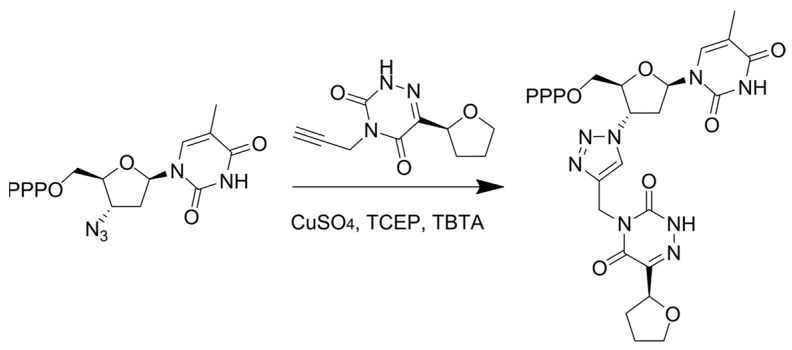
“Click” reaction used to obtain azauracil derivative AZT conjugate.

In order to avoid the removal of the obligate terminator through pyrophosphorolysis, an approach that involves conjugating an obligate terminator with an agent that is capable of covalently binding to the catalytic site of the polymerase was developed. To achieve this goal, bifunctional conjugates were obtained using “click” chemistry to modify the alkyne-bearing azauracil derivative (Figure 3) [44]. Azauracil can covalently bind near the catalytic center of a reverse transcriptase (RT) by reacting with the nucleophilic groups of amino acid residues. This was demonstrated by labeling the RT with an alkyne-containing azauracil derivative followed by a “click” reaction with fluorescent rhodamine azide and a mass spectrometry analysis of the peptides obtained after azauracil labeling and trypsin cleavage of the modified RT. Although the azauracil derivative is not capable of inhibiting the enzyme activity, the conjugate with azidothymidine (AZT) inhibits the RT activity with an EC_50_ of 12 nM, which is 23 times lower than that of the original AZT. While the incorporated obligate terminator AZT-MP can be removed through pyrophosphorolysis, releasing the polymerase and allowing for the chain synthesis to continue, the conjugate that is covalently bound to the RT near its active site blocks chain elongation and the enzyme dissociates from the chain, preventing further activity. Thus, the use of bifunctional conjugates that provide covalent binding to the target represents a promising new strategy to enhance the efficiency of obligate NAs.

### 2.2. Non-Obligate Termination

Efficient chain terminators with non-obligate MoA were also developed. In this case, the sugar residue is modified in such a way that after entering the catalytic site and/or being incorporated into the growing chain, it hinders the translocation of the polymerase and/or the entry and binding of the incoming nucleotide.

#### 2.2.1. Translocation Inhibition

**Figure 4:**
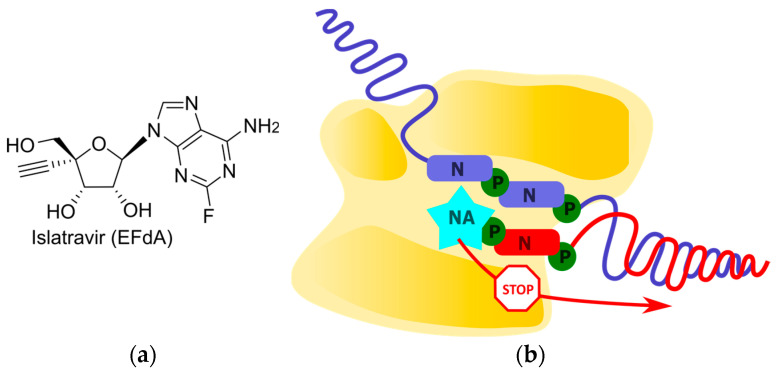
Pseudo-obligate chain terminator. (**a**) Chemical structure of islatravir (EFdA). (**b**) Schematic representation of translocation inhibition.

Islatravir (EFdA) is the first translocation inhibitor among nucleoside reverse transcriptase inhibitors (NRTIs) [45]. Due to its high similarity to endogenous nucleotides, islatravir is efficiently metabolized by cellular kinases into its active triphosphate (Figure 4). EFdA-TP tightly binds to the RT catalytic site. According to molecular modeling experiments and an analysis of resistant mutations, the 4′-ethynyl group favorably localizes in a hydrophobic pocket formed by residues A114, Y115, F160, M184, and D185 [46]. Additionally, this substituent provides a “north” conformation of the sugar residue, which is also favorable for binding [47]. These structural features allow for EFdA-TP to interact tightly with the RT catalytic site, regardless of its incorporation into the chain. As a result, the translocation of RT is hindered, preventing the efficient binding and incorporation of the next NTP. Non-obligate terminators that stop chain growth immediately after its incorporation are referred to as pseudo-obligate chain terminators. Notably, the unique mechanism of termination for RT inhibitors has allowed for the inhibition of replication in HIV strains that are resistant to previously developed NRTIs [48]. In addition, due to the mechanism involving binding in the RT catalytic site, islatravir has a high barrier to resistance as corresponding mutations reduce the viral replication efficiency [49]. Thus, islatravir maintaining the 3′-hydroxyl and introducing the 4′-ethynyl group demonstrated an inhibition of HIV RT and overcame resistance to previously developed antiretrovirals.

#### 2.2.2. Arrest of Chain Elongation through the Disruption of H-Bond Network between Polymerase and Incoming NTP

**Figure 5:**
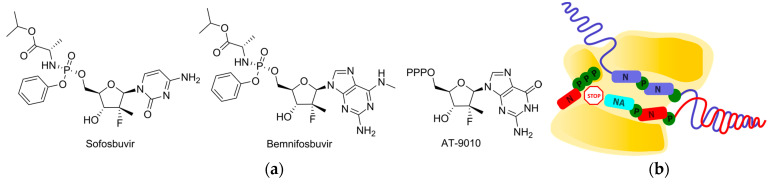
Non-obligate chain terminators. (**a**) Chemical structures of sofosbuvir and bemnifosbuvir. (**b**) Schematic representation of impairing of NTP binding.

Another promising class of non-obligate inhibitors is 2′-methylated NAs. The most successful examples are sofosbuvir and bemnifosbuvir, which are prodrugs of 2′-CH_3_-2′-F-analogues of uridine and guanosine, respectively (Figure 5). Fluorine is often used as an isosteric replacement since it is very close in size to a hydrogen, but is also similar in electronegativity to the hydroxyl group found in ribose-based nucleoside, which may facilitate recognition by viral RdRp [50,51]. The binding of UDP, 2′-CH_3_-UDP, and 2′-CH_3_-2′-F-UDP with the catalytic site of HCV RdRp was investigated using X-ray crystallography [52]. No significant differences were observed between the UDP and 2′-CH_3_-UDP positions, but several important features were found for 2′-CH_3_-2′-F-UDP, which models the active form of sofosbuvir. Its incorporation into the RdRp catalytic site disrupts the H-bonding network naturally occurring during ribonucleotide incorporation. Specifically, residue S282, which normally forms an H-bond with the 2′-hydroxyl, remains in the same position as in the apo-enzyme. However, due to the interaction between 2′-F and residue N291, the nucleotide analogue adopts a conformation that is suitable for incorporation into the growing strand. All of these interactions contribute to the inhibition of HCV RdRp via sofosbuvir as a non-obligate chain terminator. A subsequent study on the localization of the bemnifosbuvir active metabolite (AT-9010) in the catalytic center of coronavirus RdRp also demonstrated a disruption of the H-bonding network [53]. According to cryo-EM data, incorrect positioning of the incoming NTP was observed due to repulsion between its polar 3′-hydroxyl and hydrophobic 2′-CH_3_ group of incorporated AT-9010. This unfavorable position prevents the incorporation of the next nucleotide, leading to chain termination. It should be noted that sofosbuvir has a high resistance barrier and is active against several HCV genotypes [54]. Bemnifosbuvir was initially developed as an improved analog of sofosbuvir, but its ability to inhibit coronavirus RdRp has attracted more attention [55]. However, sofosbuvir does not inhibit coronavirus replication. The reasons underlying this phenomenon have not yet been revealed. It is known that HCV RdRp more effectively removes pyrimidine analogs [56]. The possibility of similar discrimination should be investigated in the case of coronavirus RdRp. The influence of including poliovirus RdRp in viral RNA 2′-CH_3_-AMP was also studied. A solution-state NMR analysis showed that the embedded NA does not hinder the binding of the next NTP, as in the case of sofosbuvir, but inhibits the conformational transition of the enzyme that is necessary for the incorporation of the incoming NTP into the growing chain [57]. Thus, MoA and the spectrum of activity of 2′-methylated NAs differ depending on the nucleobase type and the presence of 2′-F, but a success in efficient drug development hopefully will stimulate further research in this area.

#### 2.2.3. Polymerase Backtracking

**Figure 6:**
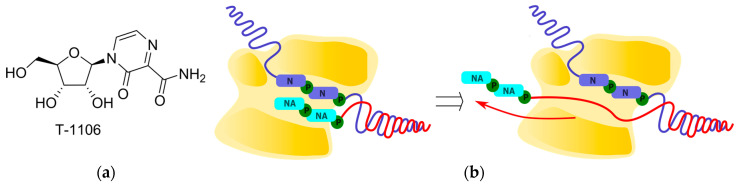
Polymerase backtracking. (**a**) Chemical structures of T-1106. (**b**) Schematic representation of polymerase backtracking.

**Figure 7:**
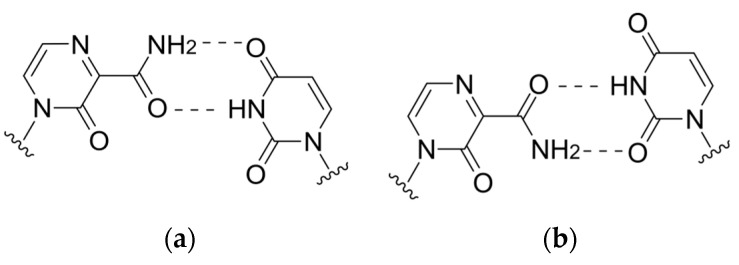
T-1106:U base pairing: (**a**) canonical and (**b**) wobble.

The violation of viral polymerase translocation was also demonstrated by T1106, which can act as an analog of any of the purine bases. According to the cryo-EM data, when incorporated by influenza virus polymerases into the growing chain, a tandem of two T1106 molecules exhibits polymerase backtracking, with the release of several last-inserted nucleotides (including both T1106) into the nucleotide entry channel, leading to RNA synthesis termination (Figure 6) [58]. Meanwhile, a single incorporation of T1106 opposite UMP in the growing strand leads to the formation of a wobble pair T1106:U at the i + 1 position of the polymerase (Figure 7), a phenomenon whose potential for the polymerase inhibition remains to be investigated.

### 2.3. Delayed Termination

**Figure 8:**
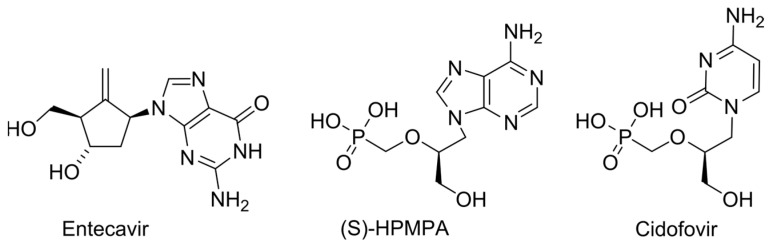
Previously developed delayed chain terminators.

Non-obligate NAs might be removed by RdRp through pyrophosphorolysis or the exonucleolytic activity [56,59]. In 2005, delayed chain termination was proposed as an effective tool to avoid the exonucleolytic removal of NAs [60]. Since then, several drugs acting through this mechanism have been developed. As a rule, these NAs possess the locked sugar conformation. Delayed chain termination supposes the inhibition of chain elongation after the incorporation of two or three nucleotides after NA is embedded due to the displacement of the growing chain in the catalytic site and the repulsion between the NA and amino acid residues in the translocation track of the viral polymerase. By virtue of steric hindrances, these NAs often provide the inhibition of chain elongation being incorporated into the template strand. Entecavir, HBV, and HIV RT inhibitor is the classic and most successful example (Figure 8) [61,62]. Surprisingly, acyclic nucleoside phosphonates such as (*S*)-HPMPA and cidofovir can also act through delayed chain termination and template-dependent replication inhibition, but their MoA differs from the aforementioned version in detail (Figure 8) [63]. Particularly, the mechanism of template-dependent inhibition is based on the exonucleolytic removal of NMP, complementary to NA-MP, due to its recognition as a DNA lesion [64].

Because islatravir has a locked “north” sugar conformation, it can be assumed that it acts through delayed chain termination. In fact, despite the elongation arrest that is usually demonstrated by islatravir, in some sequence contexts, the incorporation of an additional NTP was observed. However, repulsion between the 4′-ethynyl group and residues of the polymerase catalytic center led to the dissociation of the polymerase from the growing chain, preventing further DNA synthesis [45].

**Figure 9:**
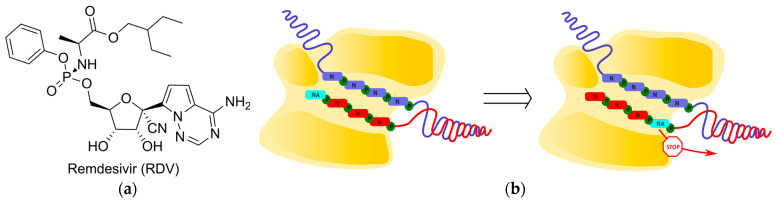
Remdesivir. (**a**) Chemical structures. (**b**) Schematic representation of delayed chain termination.

Remdesivir (RDV), a recently developed delayed chain terminator, is a prodrug of adenosine containing a 1′-cyano group, that can impede the translocation of coronavirus polymerases (Figure 9) [65,66]. RDV-TP is incorporated by coronaviral polymerases into the growing chain more efficiently than endogenous ATP, as determined by steady-state kinetic measurements. According to the molecular modeling data, this is attributed to the cyano group’s insertion into the RdRp pocket between thymine in the opposite strain and A688 in the protein. After the incorporation of RDV-MP into the growing chain at position i, an additional three nucleotides are added, followed by the termination of viral RNA synthesis at position i + 3. The molecular modeling data suggest that the repulsion between the RDV cyano group and S861 in the protein leads to the inhibition of the polymerase transition to position i + 4. This is supported by the observation that the efficiency of termination is reduced upon the mutation of S861G. However, an increase in the concentration of the incoming NTP at position i + 4 overcomes steric hindrance, allowing for viral RNA synthesis to continue. In this case, viral RNA containing RDV-MP is synthesized and can serve as a template for the production of daughter RNA strands.

**Figure 10:**
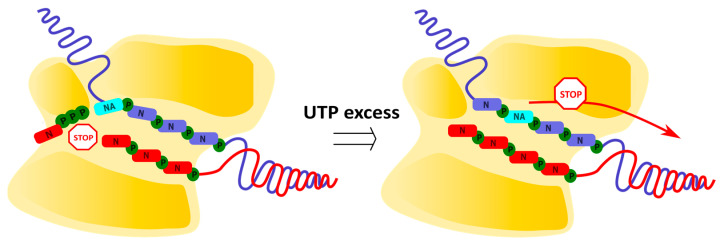
Polymerase inhibition by active metabolite of RDV incorporated in template strand.

RDV also can inhibit viral genome replication after being incorporated into the template strand of viral RNA (Figure 10) [67,68]. The presence of RDV-MP in the template strand inhibits the incorporation of complementary nucleotides into the growing chain. According to the molecular modeling data, the cyano group of the embedded RDV-MP in the template strand unfavorably interacts with A558, causing a distortion of the RMD-MP position in the catalytic site and rendering H-bonding with an incoming UTP ineffective. This is indirectly supported by the observation that the mutation of the neighboring residue V557L reduces the termination efficiency. Furthermore, further translocation of the polymerase is blocked by repulsion between the cyano group of RMD-MP and T687. In this case, the inhibitory effect of RDV-MP within the template strand can also be overcome by increasing the concentration of incoming NTPs, but the required concentration is higher than in the case of RDV-MP in the growing chain.

The inhibitory effects of RDV-TP against a panel of RNA polymerases derived from diverse RNA virus families were later studied [69]. The ability of RDV-TP to be incorporated into the growing chain by the polymerases strongly correlates with the level of antiviral activity. At the same time, the template-dependent inhibition of RNA synthesis opposite the embedded RDV-MP was uniformly recorded with all polymerases, highlighting the importance to improve the rates of prodrug metabolite incorporation while maintaining the inhibitory effects of a bulky 1′-modification.

Thus, RMD represents a prominent example of a chain-terminating NA with a dual MoA, manifested by the disruption of the polymerase translocation in the presence of RMD in both the growing and template strands. A recent study expanded an RDV activity profile and showed that there was no antagonistic effect with favipiravir [70], thus confirming their different mode of action.

### 2.4. Lethal Mutagenesis

**Figure 11:**
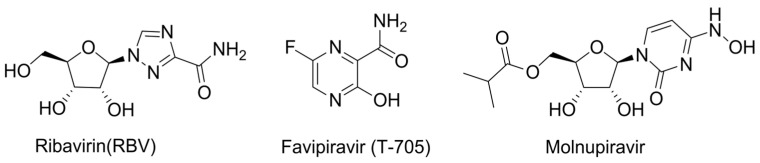
NAs acting through lethal mutagenesis.

Examples of chain termination by embedded NAs were considered above. However, some NAs are capable of inhibiting viral genome replication without inducing chain termination. They possess the ability to engage in alternative base pairings and, when present in the template strand, induce mutations in the synthesized strand. Upon surpassing a threshold, the accumulation of mutations leads to an error catastrophe and a reduction in the viral infectivity. This mechanism is referred to as lethal mutagenesis (Figure 11) [71]. The advantage of the corresponding compounds is that, being non-terminators, they are less likely to be removed due to the exonuclease activity of RdRp [34].

**Figure 12:**
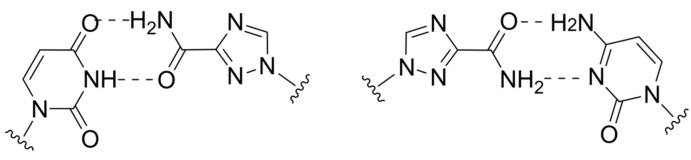
Ribavirin’s ambiguous base pairing.

The first example with this MoA is ribavirin (RBV), which contains a rotatable carboxamide group and can mimic both guanosine and adenosine (Figure 12) [72]. However, the presence of multiple MoAs for RBV makes it difficult to evaluate a contribution of lethal mutagenesis to antiviral efficacy.

**Figure 13:**
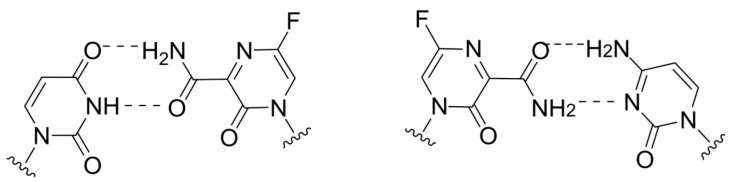
Favipiravir’s ambiguous base pairing.

The next important milestone was the development of favipiravir, which also contains a carboxamide group and is capable of alternative pairing with pyrimidine nucleobases (Figure 13). It has three times the mutation generation rate compared to RBV and a lower toxicity. However, its effectiveness is limited by low bioavailability due to rapid renal excretion, which complicates the assessment of in vivo efficacy. Additionally, the possibility of generating a favipiravir-resistant strain without reducing infectious activity was demonstrated [73]. Therefore, despite all the advantages compared to RBV, favipiravir still leaves room for modifications to improve its properties.

**Figure 14:**
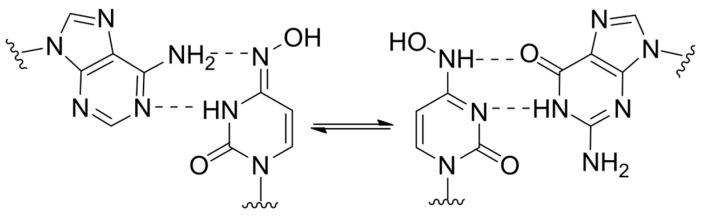
Molnupiravir’s ambiguous base pairing.

Molnupiravir, the prodrug of N-hydroxycytidine, is currently considered the most effective lethal mutagen known. It can pair equally effectively with both guanosine and adenosine (Figure 14) [74]. Unlike the aforementioned NAs, alternative pairing is achieved through tautomeric transition between amino-hydroxy and imino forms. Molnupiravir induces minimal changes in the polymerase catalytic site, making it resistant to exonucleolytic removal and causing it to have a high resistance barrier. However, there is no consensus on its ability to induce mutations in the host cell that limits its potential for broad application [75].

Overall, lethal mutagenesis is an effective strategy to combat RNA viruses. However, it is associated with high risks of the emergence of resistant strains due to the increase in genome variability and the induction of mutations in the host cell, which, theoretically, could lead to carcinogenesis and teratogenicity [34].

### 2.5. RdRp Inhibition without NA Incorporation

Nucleotide analogs are also capable of inhibiting polymerases without being incorporated into the growing chain. Cryo-EM studies of the complex between the aforementioned bemnifosbuvir and coronavirus RdRp revealed the binding of bemnifosbuvir to the Nidovirus RdRp-associated nucleotidyltransferase domain (NiRAN) [53]. Presumably, the NiRAN site may function in the synthesis of a dinucleotide primer for the polymerase (protein priming) and be involved in capping and ligation [76]. The localized AT-9010 at this site was metabolized to the corresponding diphosphate and tightly bound to this site in a flipped conformation and much more tightly relative to the endogenous substrate conformations. In addition to cryo-EM, an HPLC analysis of AT-9010 incubated with RdRp and a thermal shift assay were used to confirm binding in the diphosphate form. It was shown that in the presence of AT-9010, the attachment of UMP to nsp9 is inhibited, demonstrating the inhibition of NiRAN functions by bemnifosbuvir. It is possible that the presence of an additional MoA enables bemnifosbuvir to inhibit coronavirus replication.

Examples of non-incorporating inhibitors of viral RdRp include pyrrolopyrimidinone-based NAs [77]. The inhibitory action on coronavirus RdRp is only observed upon pre-incubation RdRp with NAs, while an inhibitory effect is not observed when NAs are added to the in vitro-assembled RdRp:RNA complex. The localization of the compound on RdRp in this study could not be determined.

The genomes of certain DNA viruses do not encode polymerases, so they utilize host cell DNA polymerases for genome replication. The selectivity of action of incorporating NAs described above in the respective sections is based on the greater substrate specificity of host cell polymerases compared to viral ones. Obviously, this strategy is not effective for viruses that use host cell polymerases. However, in this case, the prevention of interaction between the host DNA polymerase and viral genome is possible, and non-incorporating NAs can help with this. For example, polyomaviruses use the Tag protein, which binds to viral dsDNA and initiates its replication via cellular polymerases. HDP-CDV was found to be localized to the origin-binding domain of Tag, preventing replication initiation and inhibiting polyomavirus replication [78].

## 3. Inhibition of Viral Genome Methylation

In addition to polymerases, the genomes of several RNA viruses encode methyltransferases [79]. These enzymes are responsible for capping viral RNA to ensure similarity between the viral RNA and eukaryotic endogenous mRNA. This allows viruses to evade the host cell’s immune response, enhance the mRNA translation efficiency, and confer stability. Furthermore, viral methyltransferases are highly conserved, making them promising targets for the development of antiviral agents. However, the similarity of catalytic sites between eukaryotic and viral methyltransferases complicates the development of safe inhibitors.

**Figure 15:**
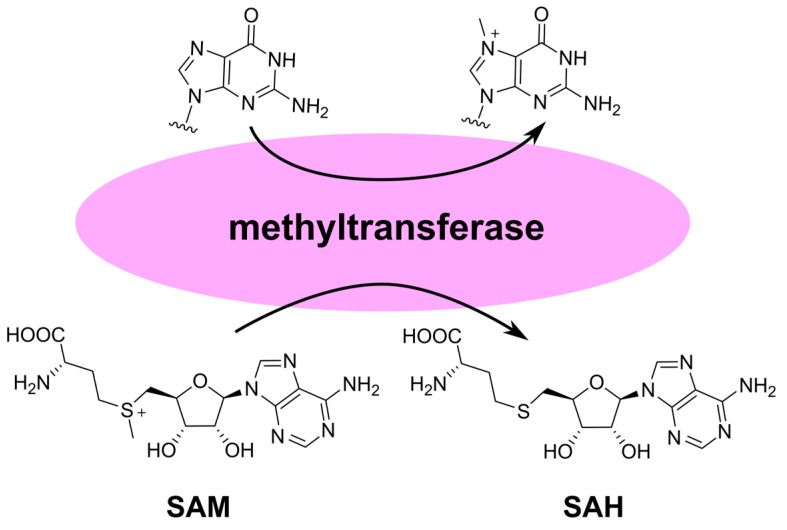
Transformation of substrate and cofactor during the guanosine-N7-methyltransferase activity. SAM—S-adenosylmethionine; SAH—S-adenosylhomocysteine.

The key steps in cap formation include the addition of guanosine to the 5′-end of mRNA and its methylation at the N7 position [80]. This structure is referred to as cap 0 and is necessary for attracting the translation initiation factor eIF4E. Animal cells also undergo methylation of one or two residues following guanosine at the 2′-OH position, resulting in cap 1 and cap 2, respectively. Depending on the virus, viral methyltransferases can facilitate the formation of both cap 0 and cap 1. In both viral and eukaryotic enzymes, methylation is coupled with the hydrolysis of the cofactor S-adenosylmethionine (SAM) to S-adenosylhomocysteine (SAH) (Figure 15). Therefore, the inhibitors of viral methyltransferases should be sought among guanosine and adenosine analogs.

**Figure 16:**
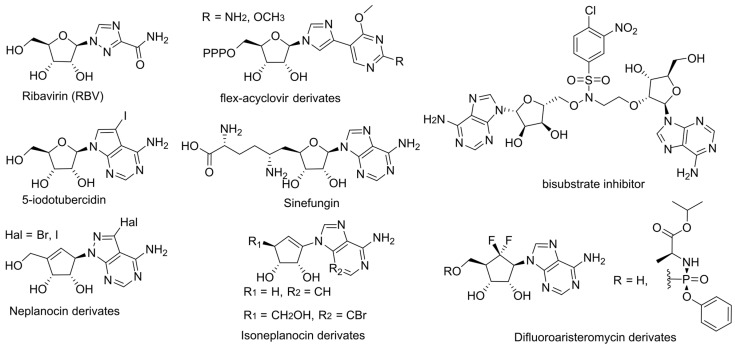
MT inhibitors.

Among guanosine analogs, RBV is capable of inhibiting viral RNA capping by occupying the GTP binding site of both viral enzymes and endogenous eIF4E, leading to a reduction in the efficiency of viral RNA capping (Figure 16) [81,82,83,84]. Recently developed flex analogs of acyclovir have shown a greater inhibition of flavivirus methyltransferase by an order of magnitude compared to the endogenous enzyme [85]. According to the molecular modeling data, it is the flexible structure of the base that provides selectivity. However, along with submicromolar activity, these compounds exhibit micromolar toxicity.

Among SAM/SAH analogs, sinefungin has long attracted the most attention for its ability to effectively bind to viral methyltransferases (Figure 16). According to the molecular modeling data, it can mimic the transition state in the methyl transfer reaction [86]. However, sinefungin itself and its derivatives do not possess sufficient selectivity [87]. It was proposed that the problem should be addressed by developing bisubstrate inhibitors that simultaneously occupy the RNA- and SAM-binding sites (Figure 16) [88]. The obtained compounds inhibit the coronavirus N7-methyltransferase more than 400 times more effectively than the eukaryotic enzyme. They represent a promising scaffold for the development of antiviral agents. Among other adenosine analogs, 5-iodotubercidin was identified as an effective inhibitor of alphavirus methyltransferase (Figure 16) [89]. Additionally, a new convenient method for evaluating methyltransferase inhibition efficacy using capillary electrophoresis was developed.

The traditional approach to the indirect inhibition of viral methyltransferases involves targeting S-adenosyl-L-homocysteine hydrolase (SAHH) [21]. SAH, the product of methylation reactions, can inhibit methyltransferases through negative feedback loop accumulation. The SAH intracellular concentration depends on the SAHH activity, so the inhibition of the enzyme can facilitate viral methyltransferase inhibition. The advantage of this approach is the potential for a broadened activity spectrum. Known inhibitors of SAHH include aristeromycin A and neplanocin A. They effectively inhibit the replication of several RNA viruses but exhibit high toxicity. Recently developed 6′-fluorinated derivatives of aristeromycin A demonstrated increased inhibitory efficacy, an expanded spectrum of activity, and reduced cytotoxicity compared to the parent aristeromycin (Figure 16) [90]. Moreover, one of the phosphonate derivatives also exhibits antiviral activity, which, according to the authors, opens up the possibility of obtaining dual-targeting antivirals that target both viral RdRp and host SAHH. However, the 5′-hydroxyl group was demonstrated to be unnecessary for SAHH inhibition along with increasing toxicity, so the prospects of the approach are questionable [21]. Among the new derivatives of neplanocin A, compounds that effectively inhibit HBV replication but have no effect on SAHH were discovered (Figure 16) [91]. The target of action is currently unknown. New derivatives of isoneplanocin A of the L- and D-series were also synthesized, and their activity data also indicate the presence of an alternative target (Figure 16) [92].

Thus, methyltransferases are promising targets for the design of antiviral agents, although selective targeting approaches are still being developed. Inhibiting SAHH, despite its long history, has been less fruitful in the development of antiviral agents compared to targeting viral polymerases. Furthermore, it was shown that synefungin can inhibit viral methyltransferases more effectively than SAH [93]. This suggests that the direct targeting of viral methyltransferases is a more promising approach.

## 4. Nucleotide Pool Depletion

The replication of the viral genome can be inhibited not only through the direct targeting of viral polymerases, but also indirectly, for example, by depleting the nucleotide pool. In this case, the enzymes of the host cell are targeted, which has both advantages and disadvantages. On the one hand, it allows for a broader spectrum of activity. On the other hand, there is a risk of increased toxicity and the emergence of off-side effects.

**Figure 17:**
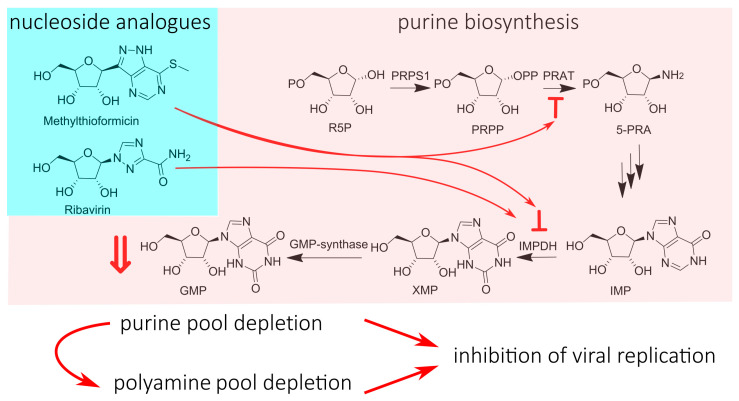
NA causes viral replication inhibition via nucleotide pool depletion. R5P—ribosyl-5-phospate; PRPS1—phosphoribosyl pyrophosphate synthetase 1; PRPP—phosphoribosyl pyrophosphate; PRAT—phosphoribosylpyrophosphate aminotransferase; 5-PRA—5′-phosphoribosylamine; IMP—inosine monophosphate; IMPDH—inosine monophosphate dehydrogenase; XMP—xanthosine monophosphate; GMP—guanosine monophosphate.

A classic example of a nucleotide-pool-depleting NA is RBV. Its mechanism involves the inhibition of the inosine monophosphate dehydrogenase (IMPDH) via RBV monophosphate (RBV-MP) [72]. The IMPDH converts inosine monophosphate (IMP) to xanthine monophosphate (XMP), which is further transformed into guanosine monophosphate (GMP) via intracellular GMP synthase, and then into GTP and dGTP (Figure 17). RBV-MP can inhibit IMPDH by acting as an IMP analog, thereby reducing the available pool of GTP for viral genome synthesis and inhibiting virus replication. Along with direct replication inhibition, GTP pool depletion increases the sensitivity of viruses to RBV’s antiviral properties due to the changed intracellular GTP:RBV-TP ratio. Subsequently, it was discovered that the depletion of the GTP pool also leads to the activation of spermidine-spermine N1-acetyltransferase 1 (SAT1), which is responsible for polyamine catabolism [94]. Polyamines play an important role in viral protein translation, virus packaging, and protease function. A depletion of the polyamine pool has been shown to have inhibitory effects on the replication of several viruses [95]. Therefore, RBV’s enhancement of polyamine catabolism can be considered an additional mechanism for inhibiting virus replication. It should be noted that RBV, which targets host cell enzymes, has a broad spectrum of activity against both RNA and DNA viruses. Additionally, it exhibits significant cytotoxicity [96]. Although caution must be exercised in drawing conclusions due to its multiple MoAs, RBV serves as a vivid illustration of the contradictions that arise when targeting host cell enzymes.

Among the regulators of purine biosynthesis currently being investigated for antiviral activity, methylthio-formycin stands out [97]. Its ability to inhibit influenza virus replication and viral polymerase activity was discovered, causing A-to-C mutations without integration into the viral genome. Methylthio-formycin is known to be an inhibitor of phosphoribosyl pyrophosphate amidotransferase (PRAT) and IMPDH, which are enzymes that play important roles in purine nucleotide synthesis (Figure 17). Therefore, methylthio-formycin is capable of depleting the pool of purine nucleotides, leading to the inhibition of virus replication. This hypothesis is supported by the fact that the addition of ATP to the cellular environment nullified the inhibitory effect of methylthio-formycin on replication. Although methylthio-formycin exhibits significant cytotoxicity, it inhibits influenza virus replication at much lower concentrations than a 50% cytotoxic concentration, allowing for its development as an antiviral agent. It is also worth noting the ability of methylthio-formycin to reduce intracellular polyamine concentrations by inhibiting their synthesis [98]. It is quite possible that methylthio-formycin may also have an additional MoA related to depleting the pool of polyamines, but this remains to be determined.

NAs are capable of depleting the pool of not only purine, but also pyrimidine nucleotides. Gemcitabine is the characteristic example due to its ability to deplete pyrimidine pool, probably via the inhibition of CTP synthase [99]. As a result, it exhausts the pool of pyrimidine nucleotides and, through an unknown mechanism, activates the transcription of interferon-stimulated genes (ISGs), resulting in the antiviral action of this NA, which will be discussed below in the corresponding chapter [100,101].

Thus, the depletion of the nucleotide pool allows for the inhibition of virus replication by targeting host cell enzymes, which predictably expands the activity spectrum but increases the toxicity. It should be noted that drugs that act through the mechanism of nucleotide pool depletion show promise for use in combination therapy with other NAs [102]. For example, the combination of the guanosine analogue INX-08189 and ribavirin, which inhibits guanosine synthesis, demonstrates synergistic effects by increasing the ratio of NA-TP (nucleoside analogue triphosphate) to NTP (natural triphosphate) in the cell, allowing for a lower dosage of both drugs [103].

## 5. Immunomodulation

**Figure 18:**
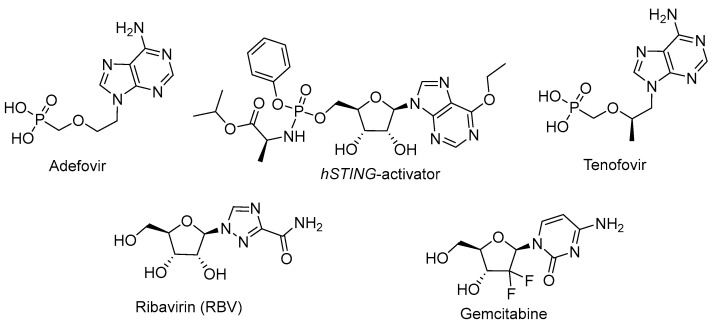
NA causes viral replication inhibition via nucleotide pool depletion.

In the fight against viral infection, the activation of the innate immune response plays an important role. Interferons are highly effective against viruses at almost all the stages of the pathogen’s life cycle [104,105]. In some cases, they are used as a component of antiviral therapy. Some NAs possess immunomodulating properties, which can significantly enhance their effectiveness. A classic example of an immunomodulating NA is RBV, which enhances the immune response by inducing a switch in the T-helper’s phenotype from 2 to 1, leading to the active production of interferons (Figure 18) [106]. According to clinical trials on anti-HBV therapies, NAs such as adefovir and tenofovir are able to induce INF-λ3 production, which further induces the transcription of interferon-stimulated genes (ISGs) and results in a reduction in HBsAg production. This is supposed to be the second MoA of these NAs (Figure 18) [107]. Gemcitibine was known to inhibit viral replication due to the depleting pyrimidine pool, enhancing the transcription of ISGs (Figure 18) [100,101]. Recently, new details on gemcitabine’s immunomodulating action were discovered, while an assessment of its prospects to be a component of hepatitis E therapy was conducted. Gemcitabine activates the phosphorylation of STAT1 regardless of JAK, triggering the activation of the interferon-sensitive response element (ISRE) and the transcription of ISGs [108]. Among the recently studied NAs, 6-ethyl purine derivatives are attracting attention (Figure 18). They activate *hSTING*, a protein that normally binds cyclic GMP-AMP produced in response to pathogen detection in cells [109]. Activated *hSTING* stimulates the phosphorylation of interferon response factor 3 (IRF3) via TANK-binding kinase 1 (TBK1). IRF3 dimerizes and translocates into the nucleus to activate interferon production and the transcription of a subset of ISGs.

Thus, stimulating interferon production is a promising additional MoA for NAs, as it allows for the innate immune system’s resources to be engaged in the fight against the virus, which is particularly useful when adaptive immunity to the pathogen has not yet been evolved.

## 6. Prodrugs

Despite the effectiveness of nucleoside analogues as antiviral agents, their application is limited by several drawbacks. Firstly, nucleoside analogues have poor solubility, and their cell penetration is limited by the efficiency of transmembrane nucleoside transporters (Figure 1) [8]. This leads to a low bioavailability of NAs. Secondly, improving NAs’ stability is necessary to enhance their efficacy and reduce toxicity. Thirdly, as mentioned earlier, NAs usually need to be phosphorylated to exhibit antiviral activity. However, phosphorylation to the monophosphate (less frequently to the diphosphate) is often a limiting step that restricts the NA efficacy [102]. Therefore, the idea of delivering masked and/or phosphorylated NAs into cells appears to be promising.

### 6.1. Phosphonates and POM/POC-Masked Prodrugs

The first step towards the intracellular delivery of monophosphates involved the development of nikavir, 3′-azidothymidine, and 5′-monophosphate [110]. However, the negatively charged phosphate group inhibits the penetration of the compound into cells and reduces its bioavailability. Another disadvantage is the possibility of phosphate reverse transformation to the parent NA via phosphatases [29].

**Figure 19:**
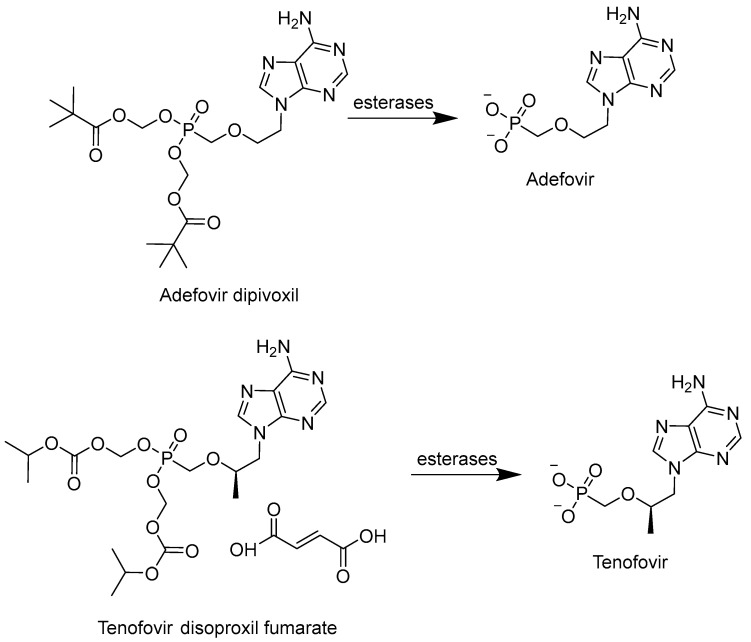
POM- and POC-modified NAs.

To avoid hydrolysis via phosphatases, a phosphonate form of NA, which implies replacing the P-O bond with a P-C bond, was developed [111]. This approach has mainly been applied to acyclic nucleotides, among which cidofovir, adefovir, and tenofovir were approved by the FDA as antiviral agents. However, the problems with bioavailability associated with the intracellular penetration of negatively charged compounds remained a challenge. To address this issue, the phosphonate group was masked with ester groups. This led to the development of adefovir dipivoxil (bis-(POM)-PMEA) and tenofovir disoproxil fumarate (bis-(POC)-PMPA), which were subsequently approved by the FDA (Figure 19) [111]. These prodrugs are metabolized to their respective monophosphates by esterases, demonstrating an improved bioavailability. In recent studies, the improvement of the pharmacokinetic properties through the development of dipivoxil- and disoproxil-masked monophosphates was shown [112]. However, this type of prodrug has a significant drawback; upon their hydrolysis by esterases, the toxic formaldehyde is produced as a byproduct, making this approach less promising for further development [113].

### 6.2. McGuigan’s ProTides

**Figure 20:**
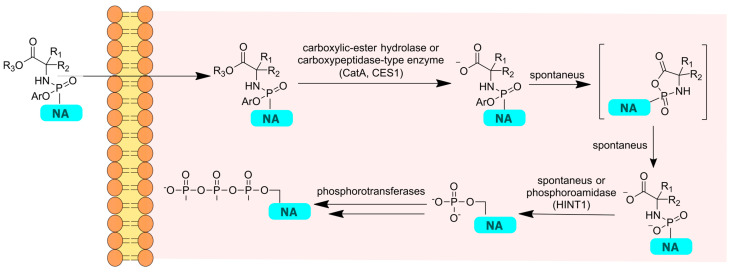
General transformation scheme of a ProTide derivative to a triphosphate form.

Another approach providing phosphorylated NAs is McGuigan’s ProTide technology based on phosphoroamidites [114]. The first work was published in 1993, and since then, this approach has been successfully applied to improve the pharmacokinetics of a number of NAs [115]. In this approach, the phosphate group is masked with two ester groups: an aromatic ester via a P-O bond and an amino acid ester via a P-N bond. Typically, a combination of benzyl and L-alanine substituents is used, although other substituents such as naphthol have proven to be more effective in some studies [116]. The resulting uncharged lipophilic molecule can penetrate the cell through passive diffusion without the need for a membrane transporter. Inside the cell, the prodrug is metabolized by enzymes with carboxypeptidase activity (usually carboxylesterases 1 or cathepsin A) and phosphoramidase activity (e.g., HINT 1) to yield the corresponding monophosphate (Figure 20).

The advantages of ProTides involve an increased bioavailability because of the enhanced transmembrane transport efficiency and improved pharmacokinetics through bypassing the first, rate-limiting phosphorylation step. In some cases, this approach allows for a reduced cytotoxicity of NA. Firstly, ProTides have a reduced risk of mitochondrial toxicity compared to the parent NA [117,118]. Secondly, when using ProTides, preferential metabolism in target organs and tissues can be achieved [119]. The mentioned enzymes are highly active in the liver, and they are the keys to the success of McGuigan’s prodrugs for HBV and HCV treatment [120,121]. However, the ability of the prodrugs to undergo efficient metabolism in the liver leads to a decreased efficacy and hepatotoxicity for the ProTide representative of RDV, whose target is the lungs [122]. Therefore, the prodrugs’ development should not be limited by McGuigan’s concept, despite ProTides’ proven effectiveness.

### 6.3. Lipid-Masked Prodrugs

**Figure 21:**
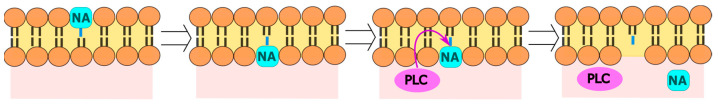
Mechanism of lipid prodrug delivery into the cell. PLC—phospholipase C.

The third interesting approach involves masking the NA phosphate/phosphonate with a lipid ether [123]. It is assumed that these prodrugs can integrate into the cell membrane similar to phospholipids and then translocate to the inner leaflet of the bilayer, where the corresponding phosphate/phosphonate is released through the action of phospholipase C (PLC) (Figure 21). This approach allows for increased bioavailability. Another advantage is the effective intestinal absorption, which facilitates the development of orally administered prodrugs. The lipid-masking strategy can also be helpful in reducing toxicity, for example, nephrotoxicity [124].

**Figure 22:**
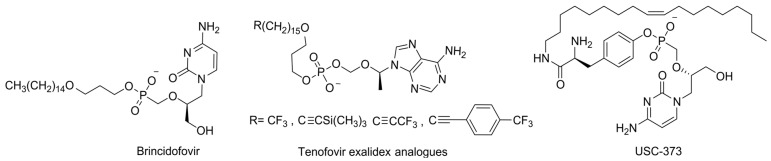
Structures of lipid-masked prodrugs.

**Figure 23:**
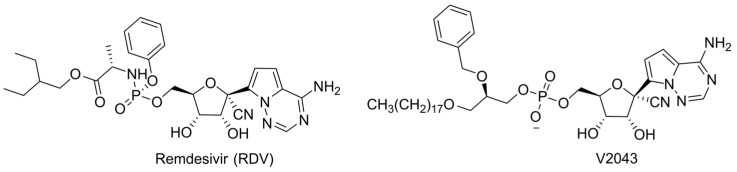
Structures of remdesivir and its lipid analogue V2043.

One of the most successful representatives is brincidofovir, which reached Phase III of the clinical trials for CMV prophylaxis in stem cell transplants and in preventing infection after kidney transplantation, and some trials targeted adenovirus (Figure 22). Although the clinical trials were terminated due to intestinal and renal toxicity and insufficient efficacy, it received approval for use in case of a poxvirus outbreak [111]. Despite having fewer successes compared to ProTide and the POM/POC-modified prodrugs, a new version of the lipid-masking approach is actively being developed. For example, a lysophospholipid analog of RDV, V2043, and its derivatives was developed (Figure 23) [122,125,126]. The idea was to obtain a prodrug for oral administration that would be more efficiently metabolized in the lungs, which enhances effectiveness and reduces drug toxicity. The lipophilic substitute promotes better absorption in the intestines. Moreover, the drug circulates mostly in the intestinal lymphatic system rather than in the portal vein, which provides greater accumulation in the lungs compared to the liver [127]. Trials on various cell lines have demonstrated equal-to-greater efficacy and reduced toxicity compared to RDV. Studies on a mouse model with V2043 showed a reduction in the viral load in the lungs when the compound was administered orally. The concept of lysophospholipid prodrugs can be considered an effective approach to obtain lung-targeted NA prodrugs for oral administration.

An increase in prodrug stability to hepatocyte metabolism should reduce hepatotoxicity. Indeed, three parameters of a lipid substitute that contribute to reducing hepatotoxicity were identified: (1) a chain length of 19–21 atoms, (2) the presence of an oxygen atom in the chain, and (3) the presence of a poorly metabolizable terminal group that impedes the oxidation of the substitute in the liver [128]. Thus, impressive improvements in the stability of the tenofovir lipid prodrug tenofovir exalidex to hepatic enzymes were achieved (Figure 22). Among lipid-based prodrugs, new polyalkyltyrosinamide prodrugs with an unsaturated chain, such as USC-373, are also attracting attention for effectively enhancing the bioavailability and antiviral activity of phosphonates; however, they are accompanied by increased cytotoxicity (Figure 22) [129,130].

**Figure 24:**
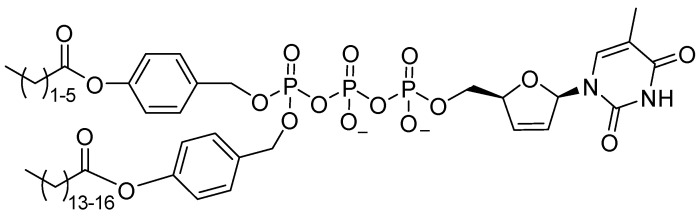
An example of masked triphosphate prodrug of 3′deoxy-2′,3′-didehydrothymidine (d4T).

In addition to monophosphate-based prodrugs, attempts were made to obtain the corresponding diphosphate and triphosphate derivatives (Figure 24) [131,132]. Although masked triphosphates that are stable at plasma pH and are capable of penetrating inside cells were developed, the enzymatic metabolism prevents the sufficient accumulation of the active form inside the cells. Thus, despite the attractiveness of the idea, its practical implementation still requires further refinement.

### 6.4. 6-Modified Purine Analogues

**Figure 25:**
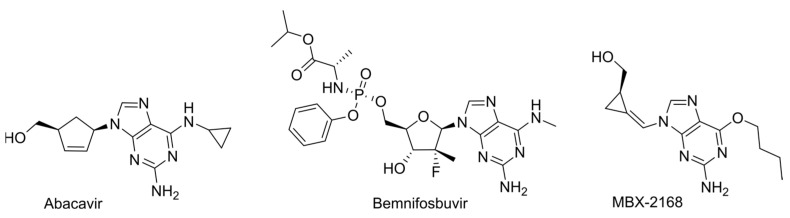
6-modified purine prodrugs.

Another approach to improving the pharmacokinetic properties is the modification of purine NAs at the 6-position. As practice shows, substitutes at the 6-position are intracellularly cleaved by deaminases, resulting in the release of the parent guanosine derivative. This strategy leads to a reduction in toxicity and an improvement in bioavailability as results of lowering the plasma concentration of the active form and increasing the prodrug lipophilicity, which promotes penetration through the cell membrane. A classic example is abacavir (Figure 25) [133]. This approach was also applied in the development of bemnifosbuvir (Figure 5).

**Figure 26:**
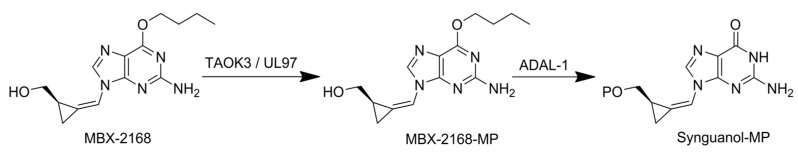
The scheme of MBX-2168 intracellular metabolism.

One of the recent and most interesting examples of 6-modified purine NAs is MBX-2168, which is a third-generation methylenecyclopropane NA designed to act against herpesviruses (Figure 25) [134]. The unique feature of herpesviruses is kinases encoded in the genome. On the one hand, it increases the efficiency of NA phosphorylation in infected cells, thus providing the possibility of safe therapy for healthy cells. On the other hand, resistant mutations often occur in viral kinases, and the difference in kinase activity among different viruses within the herpesvirus family accounts for the variations in NA effectiveness. Clinically used acyclovir and ganciclovir, as well as first- and second-generation methylenecyclopropane NAs (MCPNAs), have a limited spectrum of activity against herpesviruses. The presence of a 6-alkoxy substituent in the third-generation MCPNA MBX-2168 led to a significant expansion in the activity spectrum and an increased bioavailability. The modification enables the metabolic transformation of MBX-2168 into active singuanol-TP using endogenous enzymes, specifically TAOK3 kinase and ADAL-1 deaminase, that catalyzes the removal of the alkoxy substituent (Figure 26). On the one hand, this broadens the activity spectrum since the transformation of the analog into the corresponding monophosphate can occur independently of viral enzymes. On the other hand, the existence of a metabolic pathway independent of viral kinases provides insensitivity to mutations and, as a result, increases the resistance threshold. Thus, the strategy is suitable for expanding the range of activities and overcoming resistance.

Notably, the number of possible modifications of NAs to improve the pharmacokinetic properties is immense. The issue is discussed in more detail in the corresponding literature [21,22,111,114,135,136]. Here, the most significant approaches that allow for the enhancement of the effectiveness of NAs are listed.

## 7. Furo[2,3-*d*]pyrimidin-2-one Compounds (Bicyclic Nucleoside Analogues, BCNAs)

**Figure 27:**
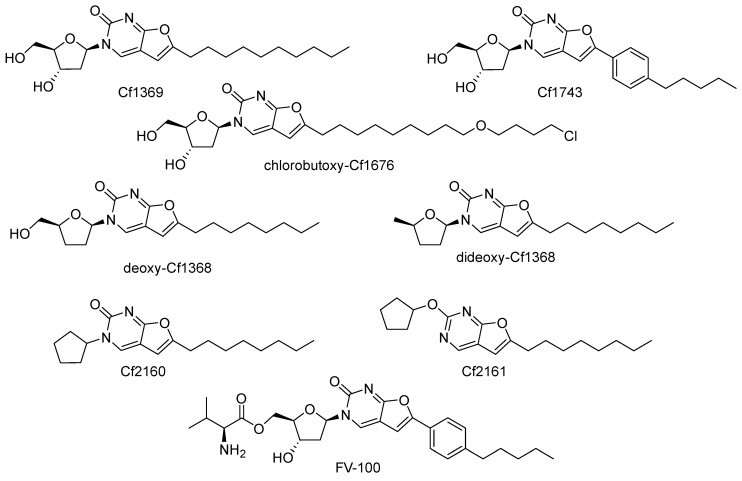
Bicyclic nucleoside analogues.

Nucleoside derivatives of Cf1369 [137] and Cf1743 types [138] (Figure 27) were serendipitously discovered by McGuigan, Balzarini et al. at the end of the last millennium to be extremely potent (subnanomolar) inhibitors of VZV reproduction with no activity against other viruses. These compounds were prepared via 5-position Sonogashira alkynylation of deoxyuridine, followed by the copper-catalyzed intramolecular cyclization of alkynyl derivatives.

Further, two decades of extensive studies on various structure variations gave rather surprising results: no structural feature improving activity was found, and Cf1743 remains the most active anti-VZV compound to date. It was shown that VZV TK (but not HSV-1 TK, HSV-2 TK, mitochondrial TK-2, or cytosolic TK-1) indeed phosphorylates BNCA compounds to mono- and di-phosphates [139]; however, their further metabolic fate remains elusive. Other furo[2,3-*d*]pyrimidin-2-one compounds, chlorobutoxy-Cf1676 [140], deoxy-Cf1368 [141], dideoxy-Cf1368 [142], Cf2160, and Cf2162 [143] (Figure 27), showed no anti-VZV activity, but showed considerable activity against HCMV. The last three compounds completely lack hydroxyls suitable for phosphorylation; therefore, their antiviral mechanism of action is definitely a non-nucleoside one. FV-100, a prodrug form of Cf1743, entered clinical trials [144,145,146]; however, it has not yet been approved due to the absence of an unequivocal confirmation that the VZV DNA polymerase is its target [147].

All antiviral BNCA compounds are highly lipophilic, and therefore should be membrane active. However, this feature of BNCTs was not studied, probably due to their narrow-spectrum antiviral activity. Moreover, the furo[2,3-*d*]pyrimidin-2-one moiety is a fluorophore emitting in the visible area. Nevertheless, to the best of our knowledge, its photochemical properties that may affect antiviral activity (e.g., ROS generation) have never been studied. Thus, after more than two decades of development, BNCAs remain mysterious potent narrow-spectrum antivirals with an unproven target and mechanism of action.

## 8. Perylene Nucleoside Derivatives Exerting Antiviral Activity through a Non-Nucleoside Mechanism

**Figure 28:**
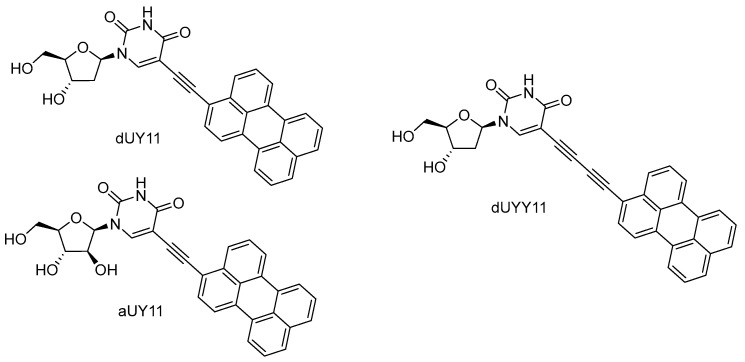
Perylene derivatives of uracil nucleosides.

5-Alkynyl-modified uracil nucleosides containing a perylene residue (Figure 28) were found to be potent broad-spectrum inhibitors of enveloped viruses. These compounds have been called rigid amphipathic fusion inhibitors, RAFIs [148]. The name refers to the structural aspects of rigidity (achieved by connecting two aromatic fragments by a triple bond) and polarity distribution (the hydrophobic perylene fragment and polar hydroxyls are located in different parts of the molecule), hinting at the key importance of these features for antiviral activity. Schang et al. [148] found a quite obvious localization of perylene compounds in the lipid membrane and hypothesized that the mechanism of their antiviral action consists of blocking the formation of a fusion pore, which should be accompanied by the transition of the positive curvature of the virion membrane to the negative curvature of the membrane in the pore. It was suggested that perylene “wedges” inserted into the virion lipid bilayer stabilize its positive curvature, thus facilitating the compound’s action as a fusion inhibitor. So, a non-nucleoside mechanism of action and the viral envelope as the target were immediately suggested for these nucleoside compounds.

Perylene nucleosides have high (IC_50_ 5–200 nM, SI > 3000) in vitro activity against various enveloped viruses—HCV, VSV, SIN, IAV, mCMV, HSV, TBEV, and ASV [149,150,151,152]. The nucleoside part of the molecule is not crucial for antiviral action; perylene derivatives lacking the sugar moiety [153] and even the uracil unit [154,155,156] are still highly active. An alternative mechanism was suggested for these compounds. Lee et al. showed the ability of dUY11 to photogenerate singlet oxygen, the need for oxygen in the atmosphere for antiviral action, the dose dependence of antiviral activity on illumination, a sharp decrease in activity in the presence of antioxidants (tocopherol and azide), and a photodependent increase in the lipid membrane rigidity [157]. Under additional illumination for 10 min, dUY11 showed subnanomolar (IC_50_ 0.2 nM) activity against HSV-1. The obtained results show an overwhelming contribution of the photochemical mechanism to the antiviral action of perylene derivatives, in fact, reducing the mechanical action (biophysical mechanism) to a negligible (and hardly measurable) effect. Recent studies confirmed the major influence of light-dependent singlet oxygen photogeneration on antiviral action [158,159]. Singlet oxygen is believed to oxidize unsaturated lipids of the viral envelope, thus disrupting its ability to fuse with a cellular membrane.

## 9. Adverse Effects

Since NAs are structurally similar to naturally occurring nucleosides/nucleotides, there is concern about their off-target effects associated with interactions with host proteins. The adverse effects may be caused by the inhibition of host nucleic acid polymerases, such as human mitochondrial DNA polymerase γ (POL γ) [160] and mitochondrial RNA polymerase (POLRMT) [161], as well as the perturbation of nucleotide metabolism/mitochondrial respiration [162] and dNTP/NTP pools [163].

**Figure 29:**
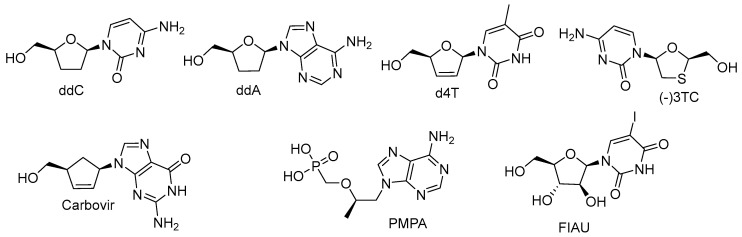
2′,3′-Dideoxy derivatives, acyclic PMPA, and FIAU bearing the 3′-hydroxyl group.

The kinetics of POL γ-catalyzed incorporation and the rate of exonuclease removal of NAs active against HIV and lacking the 3′-hydroxyl group, such as 2′,3′-dideoxycytidine (ddC), 2′,3′-dideoxyadenosine (ddA, a metabolic product of 2′,3′-dideoxyinosine (ddI)), d4T, β-L-(−)-2′,3′-dideoxy-3′-thiacytidine ((−)3TC), AZT, carbovir (CBV, an anabolite of abacavir), as well as acyclic (*R*)-9-(2-phosphonylmethoxypropyl)adenine (PMPA) and 1-(2-deoxy-2-fluoro-β-d-arabinofuranosyl)-5-iodouracil (FIAU) bearing the 3′-hydroxyl group, were studied (Figure 29) [160]. The high toxicity of d4T, ddC, and ddA resulted from 13–36-fold tighter binding compared to natural dNTP, even though their rate of incorporation was comparable to PMPA and AZT. The removal rates varied as in the following series: FIAU > (−)3TC > CBV > AZT > PMPA, d4T >> ddA (ddI) >> ddC. The high toxicity of the 2′,3′-dideoxy compounds, ddC and ddA (ddI), may result from a combination of high rates of incorporation and inefficient exonuclease removal. In turn, more efficient removal of (−)3TC, CBV, and AZT may help reduce toxicity, whereas FIAU was stably incorporated and did not block subsequent chain growth, and thus, may be mutagenic.

Ribo-NAs with activity against HCV were tested for their ability to be incorporated by human mitochondrial RNA polymerase (POLRMT) and eukaryotic core RNA polymerase II (Pol II) in vitro [161]. All of them were substrates for these polymerases and some, bearing 2′-C-methyl, 4′-methyl, and 4′-azido substituents, were also inhibitors. The proofreading activity of transcription factor S-II was capable of removing NAs from Pol II transcripts, thus explaining the relatively reduced effect on Pol II-dependent transcription in cells. In turn, the sensitivity of POLRMT lacking an active excising mechanism to NAs was confirmed, and suggests the importance of this host protein for specificity evaluation upon future drug development.

**Figure 30:**
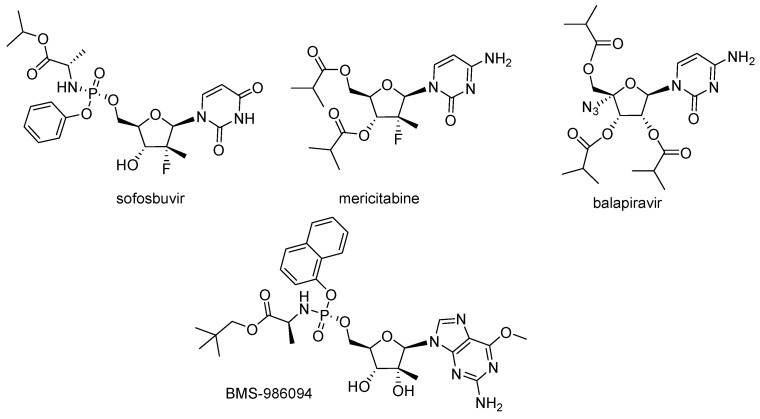
Chemical structure of sofosbuvir, mericitabine, balapiravir, and BMS-986094.

Sofosbuvir and mericitabine, as well as a number of NAs withdrawn from clinical trials, were studied for their ability to interact with human DNA and RNA polymerases and inhibit mitochondrial protein synthesis and respiration (Figure 30) [162]. NAs that were incorporated by POLRMT inhibited mitochondrial protein synthesis and showed a decrease in the mitochondrial oxygen consumption in cells. A metabolite of the prodrug balapiravir, 4′-azidocytidine, was a highly selective inhibitor of mitochondrial RNA transcription. BMS-986094, a prodrug of 2′-C-methylguanosine, showed a primary effect on mitochondrial function at submicromolar concentrations, accompanied by general cytotoxicity. In contrast, NAs containing several ribose modifications, including the active forms of mericitabin and sofosbuvir, were poor substrates for POLRMT and did not exhibit mitochondrial toxicity in cells.

The toxicity of NAs may also come from the disruption of natural NTP pools, as discussed above for ribavirin. AZT possesses a moderate inhibitory activity on POL γ [160] but is a competitive inhibitor of the thymidine kinase 2-catalyzed phosphorylation of thymidine to TMP, thus limiting the TTP pool and lowering mitochondrial DNA replication [164,165].

Remdesivir, an approved anti-SARS-CoV-2 drug, possesses weak inhibitory activity on POLRMT, leaving open the possibility of mitochondrial off-target effects and toxicity. In this regard, RDV was evaluated for mitochondrial toxicity and for the effects on mitochondrial DNA content, mitochondrial protein synthesis, cellular respiration, and the induction of reactive oxygen species [166,167]. Consistent with the reported clinical safety profile, the results demonstrate a low potential of RDV for off-target toxicity.

In addition to the mentioned host targets, abacavir exclusively binds within the HLA-B*57:01 antigen binding groove and alters the preference for the C-terminal amino acid residue of the bound peptide, leading to changes in the immunopeptidome [168,169,170]. As a result, abacavir hypersensitivity syndrome can occur in individuals expressing the HLA-B*57:01 major histocompatibility complex class I allotype.

In conclusion, the interactions of NAs with a number of cellular proteins may contribute to the toxicity that is observed. Using biochemical and cell-based assays, a preliminary assessment before entering animal toxicity studies will be helpful for reducing pre-clinical and clinical toxicity liability [163].

## 10. Conclusions

**Figure 31:**
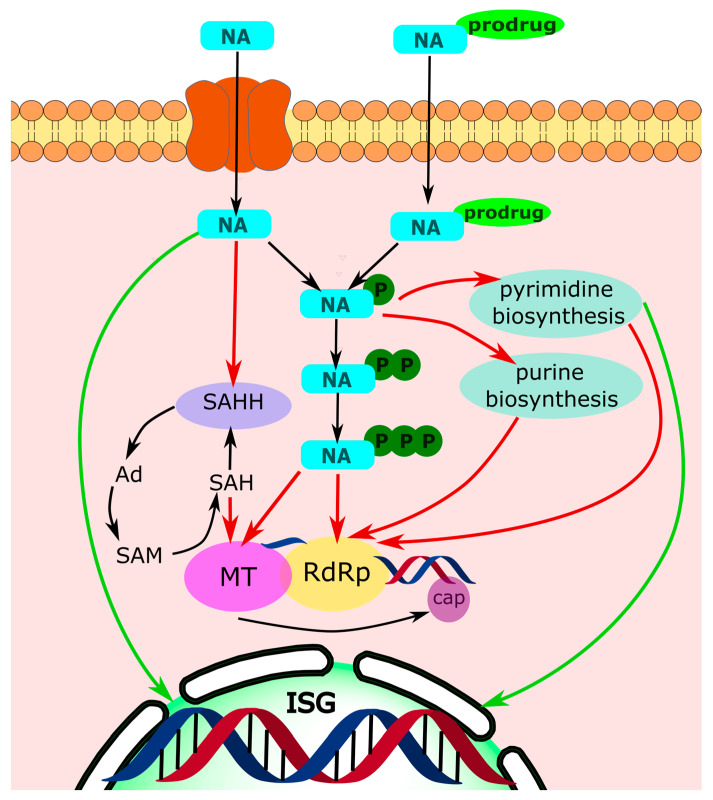
NA mechanisms. Red arrows represent inhibitory action of NAs and green ones show activation of a metabolic pathway. ISG—interferon-stimulated genes; RdRp—RNA-dependent RNA polymerase; MT—methyltransferase; SAHH—S-adenosyl-L-homocysteine hydrolase; SAH—S-adenosyl-L-homocysteine; SAM—S-adenosylmethionine; Ad—adenosine.

This review highlights the recent advances in MoA elucidation for well-known and recently developed NAs (Figure 31). Viral polymerases, particularly RdRp, that are involved in the life cycle of HCV, SARS-CoV-2, and Ebola virus, are still the most attractive targets. The combination of cryo-electron microscopy, an analysis of resistant mutations, and molecular modeling experiments allows for new MoAs to be revealed and for existing MoAs to be refined that may facilitate the development of drugs with multiple MoAs in the future.

Reducing toxicity, creating broad-spectrum agents, and overcoming resistance continue to be the main objectives. Targeting host cell enzymes can increase toxicity, while targeting viral enzymes can stimulate resistance development and narrow the spectrum of activity. However, some NAs (particularly islatravir) targeting viral polymerases require specific mutations that decrease replicative efficiency, providing a high resistance barrier. Multitargeting strategies may be considered optimal. In addition to polymerases, promising targets include methyltransferases and new viral enzymes such as DNA packaging terminase, which have not yet been targeted by NAs [171]. Involving host cell enzymes in metabolism to the active form remains a key strategy for reducing toxicity and expanding the spectrum of action. The activation of the host cell’s immune response also holds promise, although it may limit application in certain cases.

Studying the mechanistic details of NA action using powerful toolboxes can help to identify the most effective strategies for drug design. The development of safe and efficient medications helps society to defend itself against pandemic threats and improve life quality.

## Data Availability

Not applicable.
